# Abdominal unicentric Castleman’s disease: a case report

**DOI:** 10.11604/pamj.2021.38.339.28859

**Published:** 2021-04-08

**Authors:** Samir Bradai, Amal Khsiba, Abdelwaheb Nakhli, Moufida Mahmoudi, Asma Ben Mohamed, Abir Chaabane, Emna Chelbi, Medhioub Mona, Lamine Hamzaoui, Mousadek Azouz

**Affiliations:** 1 Gastroenterology Department, Mohamed Tahar Maamouri Hospital, Nabeul, Tunisia,; 2 Histopathology Department, Mohamed Tahar Maamouri Hospital, Nabeul, Tunisia

**Keywords:** Castleman’s disease, abdominal localization, surgery, case report

## Abstract

Castleman's disease is a rare disease characterized by benign lymphoepithelial proliferation. There are two forms: unicentric and multicentric Castleman's disease. Mediastinal location is the most frequent. Intra-abdominal Castleman's disease is a rare presentation. We report a case of 65-year-old female who presented with epigastric pain. Investigations revealed a retroperitoneal mass which was surgically resected. Histopathological examination showed hyaline-vascular type Castleman's disease. In conclusion, Castelman´s disease is a diagnostic challenge and it must be included in the differential diagnosis of retroperitoneal tumors.

## Introduction

Castleman's disease (CD) was first described by Benjamin Castelman in 1954 [1]. It is characterized by an angiofollicular lymph node hyperplasia. Two forms are observed: unicentric (UCD) and multicentric (MCD). Unicentric is the most common one (90%) and is generally asymptomatic [2]. Mediastinum is the most common site. The abdomen is rarely affected by this pathology [3]. We present a case of a female patient with an abdominal pseudotumor Castleman's Disease for which she underwent surgery.

## Patient and observation

**Figure 1:**
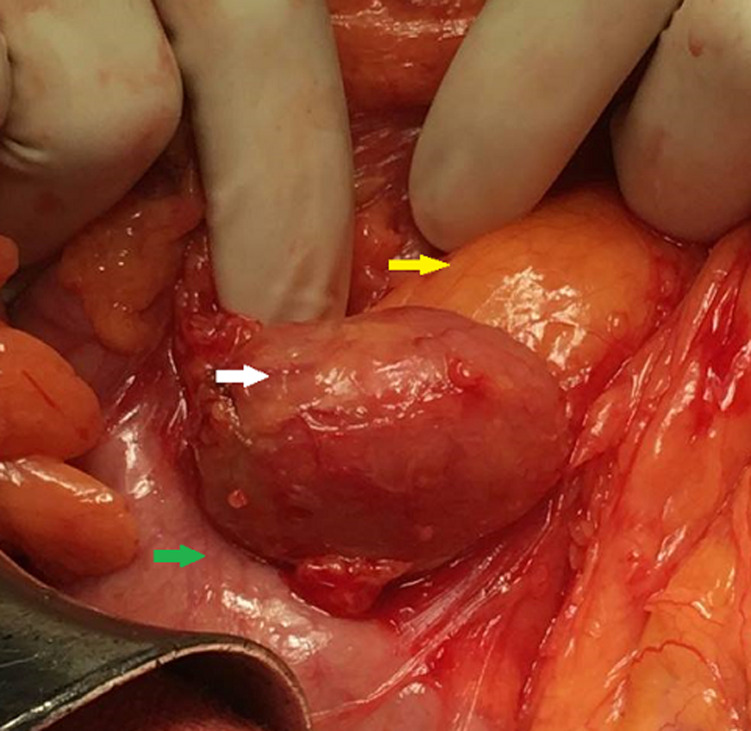
intraoperative aspect; white arrow: Castleman tumor; yellow arrow: pancreatic head; green arrow: duodenum

**Figure 2:**
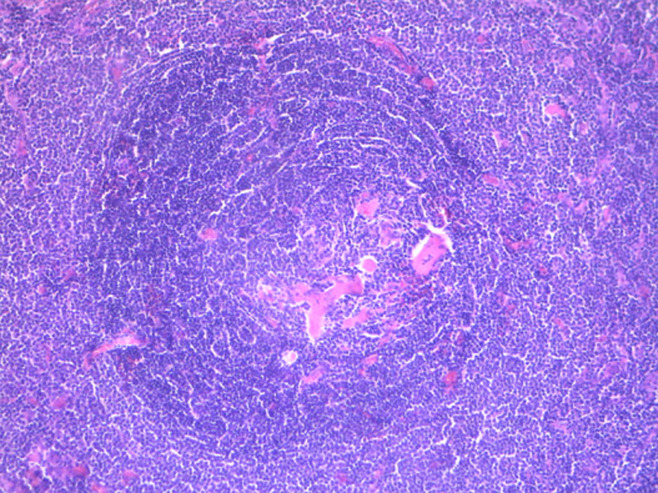
histological examination; Castleman’s disease of hyaline vascular type

**Figure 3:**
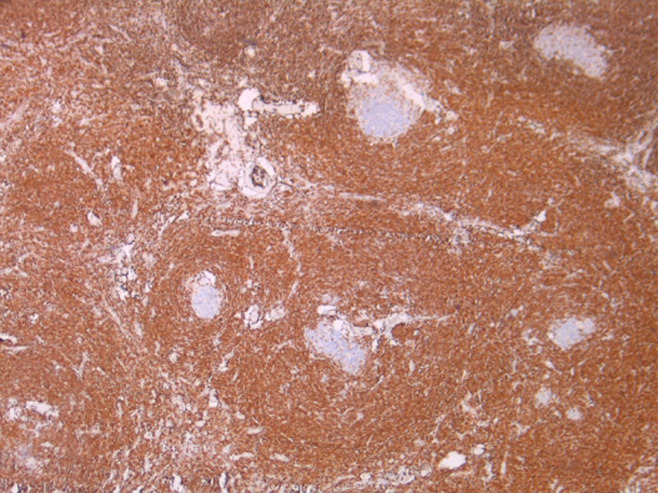
immunohistochemical staining positive for CD20+

A 62-year-old female presented with intermittent epigastric pain of one month duration. Her past medical history was significant for type II diabetes and hypercholesterolaemia. She had no family history of malignancy. Physical examination was normal. She had no cervical, axillary or inguinal lymphadenopathy. Routine blood parameters were normal. Upper gastro intestinal endoscopy was normal. Ultrasound imaging showed a homogeneously hypoechoic epigastric mass. Computerized tomography (CT) of the abdomen revealed a mass measuring 3 cm arising between the first portion of duodenum and the pancreas. Since the mass was small and retroperitoneal, we decided to proceed to mass excision. Intraoperatively, there was a well-encapsulated soft-tissue mass measuring 3.5 x 2.5 x 1.5cm, firm in consistency and deriving blood supply from multiple sources (Figure 1). Mass was excised in toto. The patient recovered without complications and was discharged within two days. Microscopic examination showed lymphoid parenchyma composed of multiple lymphoid follicles of variable size with characteristic “onion skin” layering of lymphocytes in the mantle zone. In interfollicular sectors, multiple hyaline vascular structures were noticed, giving “lollipop” appearance (Figure 2). Immunohistochemical study revealed that tumor cells were positive for CD20+ mainly in the follicular regions (Figure 3). B-cell lymphoma 2 (Bcl-2) and human herpesvirus 8 (HHV-8) antibody were negative. Human immunodeficiency virus test (HIV) was also negative. The patient is currently doing well, nine months after the surgery.

## Discussion

Castleman´s disease is rare with an incidence of 25 per million patient-years [1]. It is characterized by an angiofollicular lymph node hyperplasia. There are two presentations of CD according to the number of lymph nodes involved: unicentric and multicentric types. Unicentric Castleman´s disease is a localized disease affecting a single enlarged lymph node or a group of adjacent nodes. It is the most frequent type of CD (90% of cases). This variant is usually asymptomatic or paucisymptomatic [1,2]. Multicentric affects multiple lymph node areas. In this form, patients mostly present with fever, night sweats, weight loss, generalized lymphadenopathy and hepatosplenomegaly [1,4]. Computerized tomography scan shows a homogeneous or heterogeneous mass of soft-tissue density with rim enhancement and slow washout [5]. In unicentric Castelman´s disease, mediastinum is the most common site (70%) [3]. The abdomen is rarely affected and only few cases have been reported [3]. The retroperitoneal location, as in our case, is very rare and represents only 7% of cases [6]. Preoperative needle biopsy or fine needle aspiration is not recommended because mostly the amount of tissue is not sufficient to confirm the diagnosis. Besides, there is a possibility of spreading tumor cells and a risk of severe bleeding. Hence, the diagnosis is usually based on pathology after surgical resection [7]. Histologically, CD can be categorized into either hyaline-vascular type, plasma cell type or mixed type. Hyaline-vascular type consists of proliferation of small lymph follicles with hyalinization of its wall while Plasma cell type contains polyclonal plasma cells with a less marked hyalinization and vascularization [1,3]. Usually, UCD has a hyaline-vascular type morphology while MCD has plasma cell type or mixed type [1].

The etiology of this pathology is unknown. Chronic inflammation, immunodeficiency, autoimmunity, tuberculosis, toxoplasmosis and Epstein Barr virus have been incriminated [3]. Some multicentric forms are associated with HSSV-8 or HIV infections [8]. The role of Interleukin 6 (IL6) has been reported in the genesis of MC disease [7]. Surgical resection is the gold standard treatment for UCD [7]. In our case, the lesion was totally removed. For unresectable cases, aggressive surgical treatment is not recommended as this may increase the rates of morbidity and mortality. Mass reduction with neoadjuvant rituximab and radiotherapy can allow complete surgical resection with a lower morbidity [9]. If the resection is successful, prognosis is excellent [7]. Multicentric Castleman´s disease is more aggressive and has a poor prognosis [1]. Given that the disease is rare, there are no firm recommendations. Many agents have been tried, such as immunotherapy with monoclonal antibodies directed at IL-6 (siltuximab), antiviral agents when MCD is associated with HSSV-8 or HIV infections, and chemotherapeutic agents (doxorubicin, vincristine, cyclophosphamide, melphalan, and chlorambucil) [7]. MCD may progress to lymphoma, therefore a regular surveillance is mandatory [10].

## Conclusion

Unicentric Castelman´s disease is a diagnostic challenge and it must be included in the differential diagnosis of retroperitoneal tumors. Preoperative diagnosis is difficult due to a lack of specific radiologic markers and the poor performance of biopsy. Complete resection is the gold standard treatment for UCD. Further studies are needed to establish recommendation for the management of MCD.
